# Comparative analysis reveals the complex role of histoblast nest size in the evolution of novel insect abdominal appendages in Sepsidae (Diptera)

**DOI:** 10.1186/s12862-018-1265-3

**Published:** 2018-10-10

**Authors:** Dacotah Melicher, Kathy F Y Su, Rudolf Meier, Julia H Bowsher

**Affiliations:** 1 U.S. Department of Agriculture/Agricultural Research Station, Bioscience Research Laboratory, 1605 Albrecht Boulevard, Fargo, ND 58102 USA; 2 Department of Biological Sciences, National University of Singapore, 14 Science Dr 4, Singapore, 117543 Singapore; 3 Department of Biological Sciences, North Dakota State University, 1340 Bolley Drive, 218 Stevens Hall, Fargo, ND 58102 USA

**Keywords:** Sepsidae, Histoblast, Novelty, Evolutionary history, Comparative morphology

## Abstract

**Background:**

The males of some sepsid species (Sepsidae: Diptera) have abdominal appendages that are remarkable in several ways. They are sexually dimorphic, have a complex evolutionary history of gain and loss, and can be jointed and thus highly mobile. The sternite brushes are used extensively in complex courtship behaviors that differ considerably between species and during mating. The abdominal appendages have a novel developmental pathway developing from histoblast nests rather than imaginal discs.

**Results:**

We focus on the evolution of cell number, nest area, and segment length in both sexes to understand how this tissue relates to the formation of novel abdominal appendages. We map histoblast nest size of wandering-phase larvae of 17 species across 10 genera to a phylogenetic tree of Sepsidae and demonstrate that abdominal appendages require significant increases of histoblast nest size and cell number in most species while one species produces small appendages even without such modifications. In species with particularly large appendages, not only the nests on the fourth, but nests in neighboring segments are enlarged (*Themira biloba*, *Themira putris*). The loss of abdominal appendages corresponds to the loss of an enlarged fourth histoblast nest, although one species showed an exception to this pattern. One species that constitutes an independent origin of abdominal appendages (*Perochaeta dikowi*) uses an unusual developmental mechanism in that the histoblast nest sizes are not sexually dimorphic.

**Conclusions:**

The surprisingly high diversity in histoblast size and degree of sexual dimorphism suggests that the developmental mechanism used for abdominal appendage formation in sepsids is highly adaptable. The presence of appendages usually correlate with increased histoblast cell number and in most cases appendage loss results in a return to ancestral histoblast morphology. However, we also identify several exceptions that indicate the abdominal appendages have a malleable developmental origin that is responsive to selection.

**Electronic supplementary material:**

The online version of this article (10.1186/s12862-018-1265-3) contains supplementary material, which is available to authorized users.

## Background

The evolution of novel traits is central to understanding the diversity and disparity of organismal body plans. Despite the importance of understanding novelty and innovation, comparative study of the development of novel structures has been a historically neglected area of research [1–4]. Sexual selection has produced many examples of novel traits and traits exposed to sexual selection evolve rapidly and are morphologically diverse. This renders them good models for investigating the importance of constraint and selection. While selection is a significant driver of adaptation, particularly in sexually selected traits, genetic constraints can limit the ability of selection to drive evolution in particular directions [5–8].

Sepsid flies (Diptera: Sepsidae) are an excellent system to study novel evolutionary traits. Male sepsids in some genera possess abdominal appendages which are novel, morphologically diverse structures that consist of a moveable joint and bristles attached to a modified sternite. The appendages are a sexually selected trait used during complex and diverse courtship behaviors and during mating [9, 10]. The abdominal appendage is an evolutionarily novel structure with no known homology within Diptera [11–13], and has a complex evolutionary history of gain and loss [14]. The abdominal appendages also have a novel developmental origin from histoblast nests, which are clusters of imaginal cells that lack the complex three-dimensional organization of imaginal discs which produce limbs and other adult morphological structures. In dipterans histoblast cells develop into adult abdominal epithelium by proliferating toward segment boundaries during pupation to line the interior of the cuticle [15]. The sepsid abdominal appendage develops only from the 4th ventral histoblast nests, and while neighboring segments may possess enlarged nests, histoblast cells in all other segments develop in to epithelium. Histoblast nest location in sepsids is homologous to those in *Drosophila melanogaster* and other Dipterans and consist of three bilaterally symmetrical pairs. Histoblast cell numbers per nest in most dipterans vary between species, but there is generally no segment-specific or sexually dimorphic patterns in cell number or nest size [16, 17]. In contrast, males in some species of sepsids have enlarged histoblast nests in the appendage-producing segment. An exception is *Perochaeta dikowi,* an appendage-bearing species with enlarged histoblast nests in males and females [14].

Previous research suggested that sepsid appendages may develop using different developmental mechanisms in different species [12, 14]. In sepsids as well as dipterans broadly, histoblast cells are specified during embryogenesis and are known to remain consistent in number until the initiation of pupation when they begin to proliferate and form the adult epithelium [17]. Here, we test whether histoblast nest morphology in larvae immediately prior to cell division and proliferation is correlated with morphological diversity in the adults. This question is addressed using 17 species from across Sepsidae which allows for testing whether species that lost the trait return to the ancestral histoblast nest morphology, and whether the presence of abdominal appendages is consistently associated with a gain in the number of histoblast cells. Also, previous investigations of histoblast nest morphology did not account for differences in body size between species and did not include a representative of the sister group of all remaining sepsids (*Orygma* + *Ortalischema*), which lacks abdominal appendages. Measurements of histoblast nest area, cell number, cell nuclei size, and segment length were taken. We then use phylogenetic information in order to study the relationship between histoblast cell number and the evolution of abdominal appendages.

## Results

**Fig. 1:**
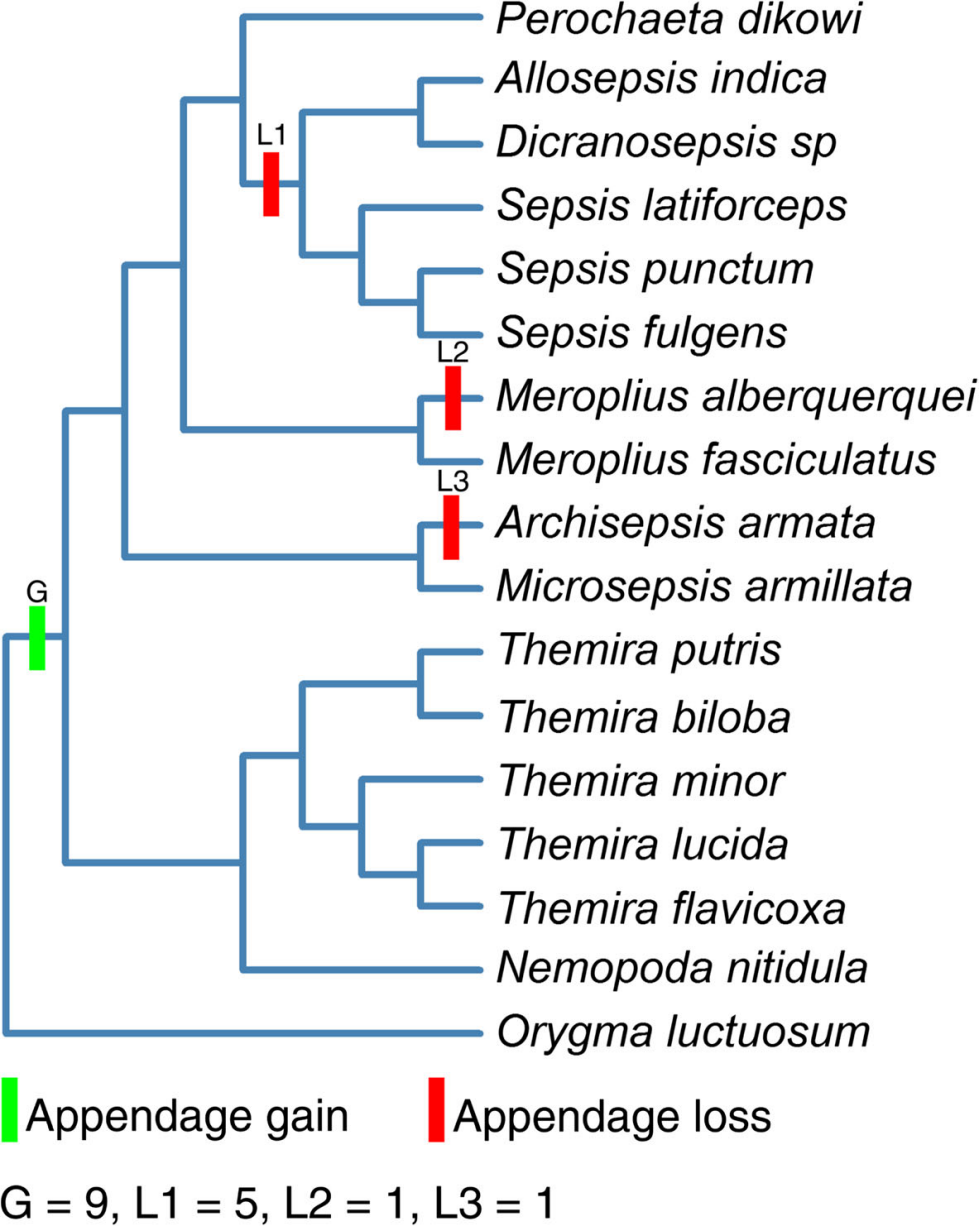
Phylogeny of sepsid species with evolutionary history of gains and losses. The current phylogeny of Sepsidae supports one gain of the appendage and multiple losses. The species sampled for histoblast analysis represent one primary gain and three independent losses of the abdominal appendage as well as *Orygma luctuosum*, a sister group to the remaining sepsid species which lacks abdominal appendages. The pattern of gains and losses was inferred from a phylogeny with more taxa [18]

**Fig. 2:**
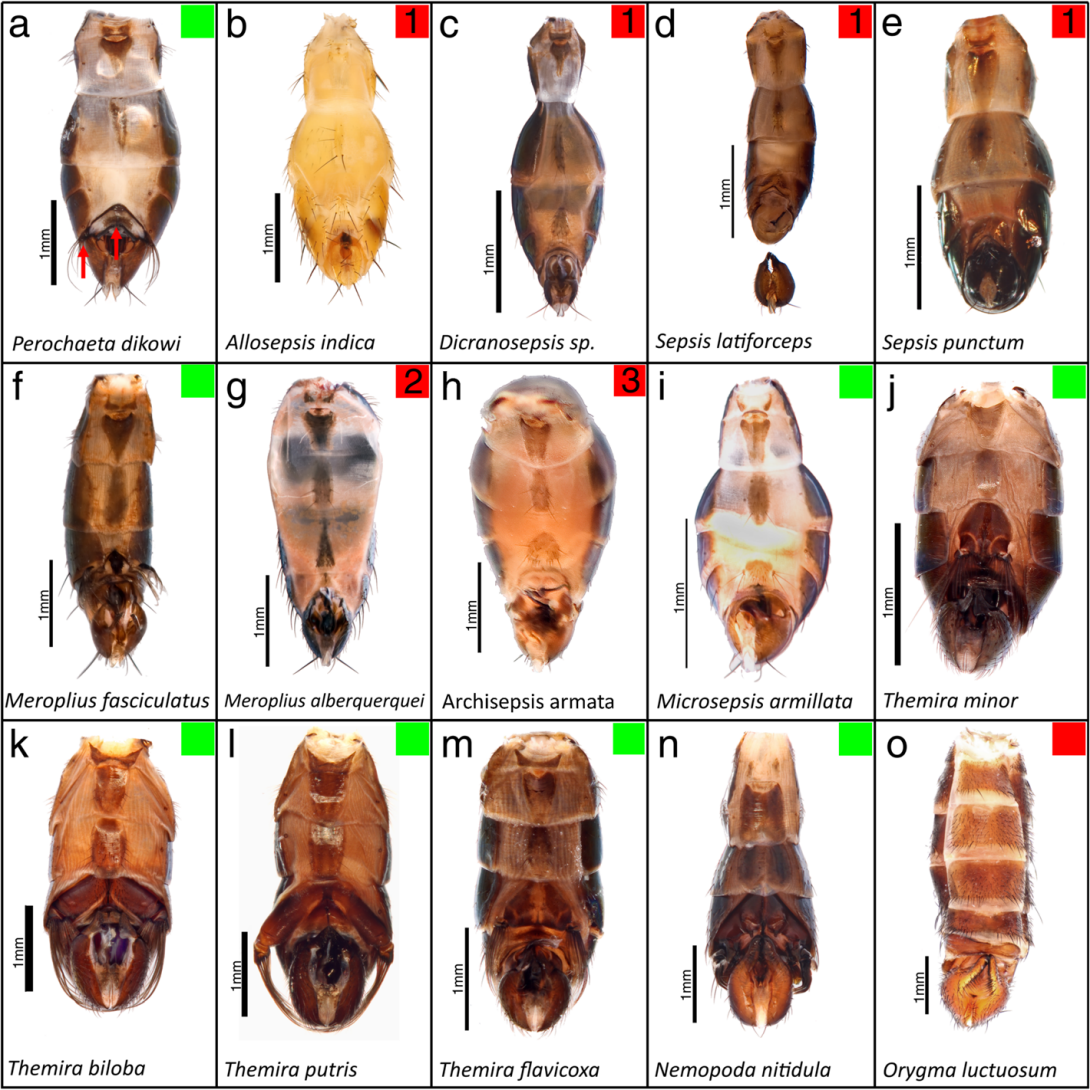
Abdominal view of adult flies of the sampled species. Species that posess the appendage are in green and species without appendages in red with independent losses indicated by number. Primary gain of the appendage (**a**, **f**, **i**-**n**) results in a modified sternite, joint and bristles as compared to the ancestral state (**o**). The current phylogeny supports three independent losses of the appendage (L1 = **b**-**e**, L2 = **g**, L3 = **h**). The degree of sternite modification and size of the appendages are highly variable between species. *T. biloba* and *T. putris* (**k**, **l**) posses extensive sternite modification and long appendage brushes relative to other species. Loss of the appendage also results in the loss of the modified sternite which returns to the ancestral state. Scale bars indicate 1 mm

**Fig. 3:**
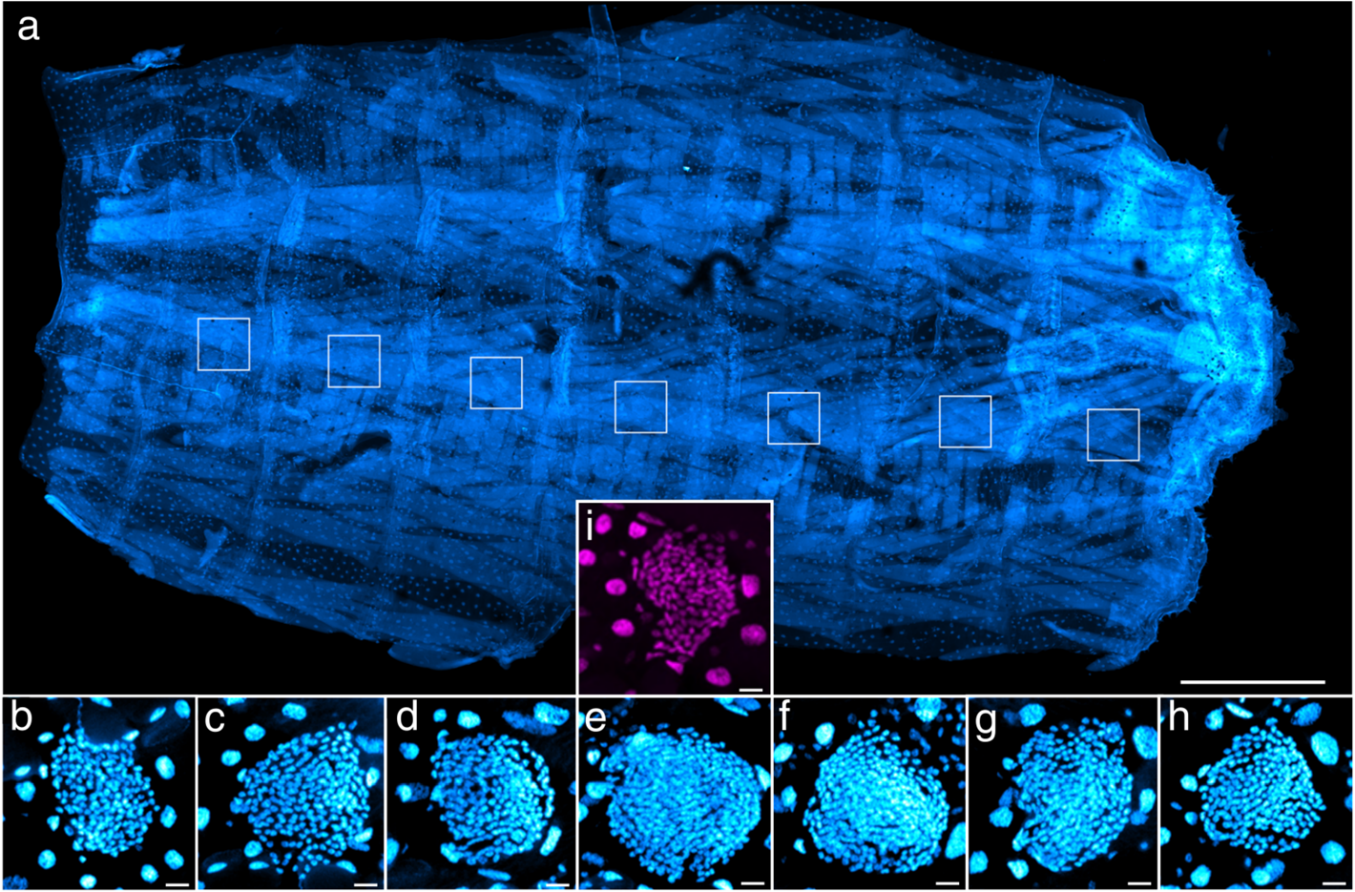
*Themira biloba* larval epidermis (**a**) with ventral histoblast nests in abdominal segments enlarged showing a sexually dimorphic increase in nest size, cell number, and cell density peaking in the 4th abdominal segment (**b**-**h**). The 4th female histoblast nest is not significantly larger than nests in other segments and is shown for comparison (**i**). Scale bars indicate 500 μm (**a**) and 25 μm (**b**-**i**)

The objective of this study was to determine if histoblast nest morphology between sexes and segments was consistent across the evolutionary history of gains and losses of the appendage. A previous study that included four species determined that histoblast cell counts have three distinct patterns which correspond to adult abdominal appendage morphology with an increase in histoblast cell number correlating with the presence of the appendage [14]. However, there is significant variation in adult appendage morphology and increased sampling may reveal additional patterns of histoblast nest morphology. The purpose of this analysis was to determine if these morphological correlations persist across a broad range of species and determine if there are other patterns or transitional states present. The species sampled include multiple appendage-bearing species that represent the primary gain of the trait. We also included species that represent three independent losses of the trait, and *Orygma luctuosum* which is an appendage-less sister taxon (Fig. 1). Adults of some species have large appendage brushes and an exaggerated fourth sternite while other species have smaller brushes and minimal sternite modifications (Fig. 2). In some species, the presence of an appendage correlates with enlarged histoblast nests that are sexually dimorphic (Fig. 3).

### Ancestral state of histoblast cell counts in Sepsidae

**Fig. 4:**
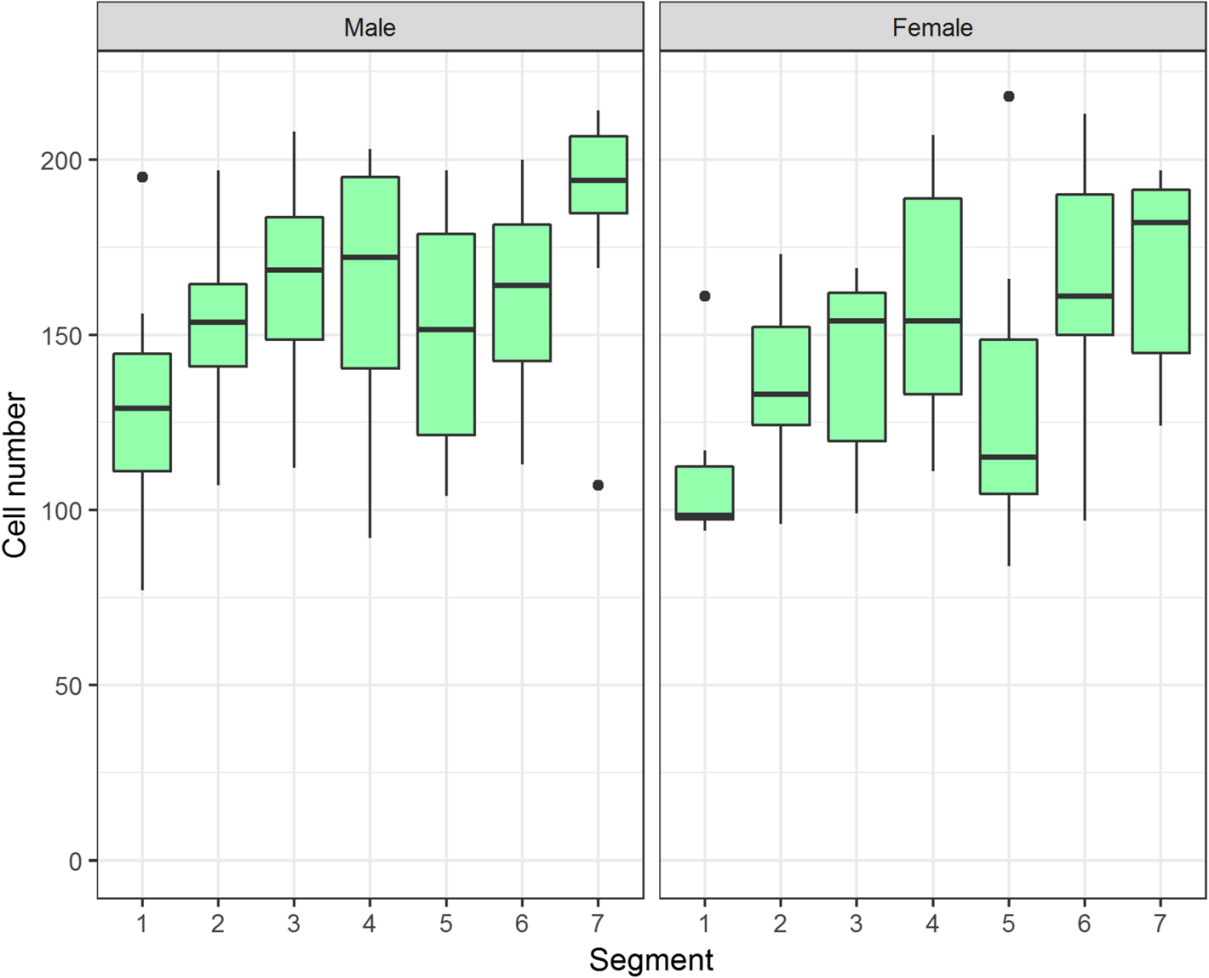
The histoblast nest in *O. luctuosum* shows no significant sexual dimorphism or increase in cell number or nest density in the 4th segment which likely represents the ancestral state. Refer to Table 3 for the number of individuals sampled

A goal of this study was to infer whether sexually dimorphic cell number preceded or coincided with the evolution of abdominal appendages in males. *Orygma luctuosum* is a species that represents the sister group to all other Sepsidae. *O. luctuosum* does not have abdominal appendages, and likely retains the ancestral state. Histoblast cell number in *O. luctuosum* were not sexually dimorphic (Fig. 4, Table 1) indicating the ancestral state of sepsids is likely a consistent number of histoblast cells across sexes that is also known from other Diptera.

**Table 1:** Appendage presence significantly increases sexual dimorphism in histoblast nests in most species

Species	Appendage status	Sex effect	Segment effect
*Orygma luctuosum*	Putatively ancestral	0.86	***
*Archisepsis armata*	Absent	0.79	0.26
*Allosepsis indica*	Absent	0.77	***
*Dicranosepsis* sp.	Absent	0.42	***
*Sepsis fulgens*	Absent	0.07	***
*Sepsis latiforceps*	Absent	0.11	**
*Sepsis punctum*	Absent	0.9	**
*Meroplius alberquerquei*	Absent	**	***
*Meroplius fasciculatus*	Present	***	***
*Nemopoda nitidula*	Present	***	***
*Themira biloba*	Present	***	***
*Themira flavicoxa*	Present	***	***
*Themira lucida*	Present	***	***
*Themira minor*	Present	***	***
*Themira putris*	Present	***	***
*Microsepsis armillata*	Present	0.82	***
*Perochaeta dikowi*	Present	0.46	***

**p < 0.01, ***p < 0.001

### Histoblast cell counts change with the gain and loss of abdominal appendages

**Fig. 5:**
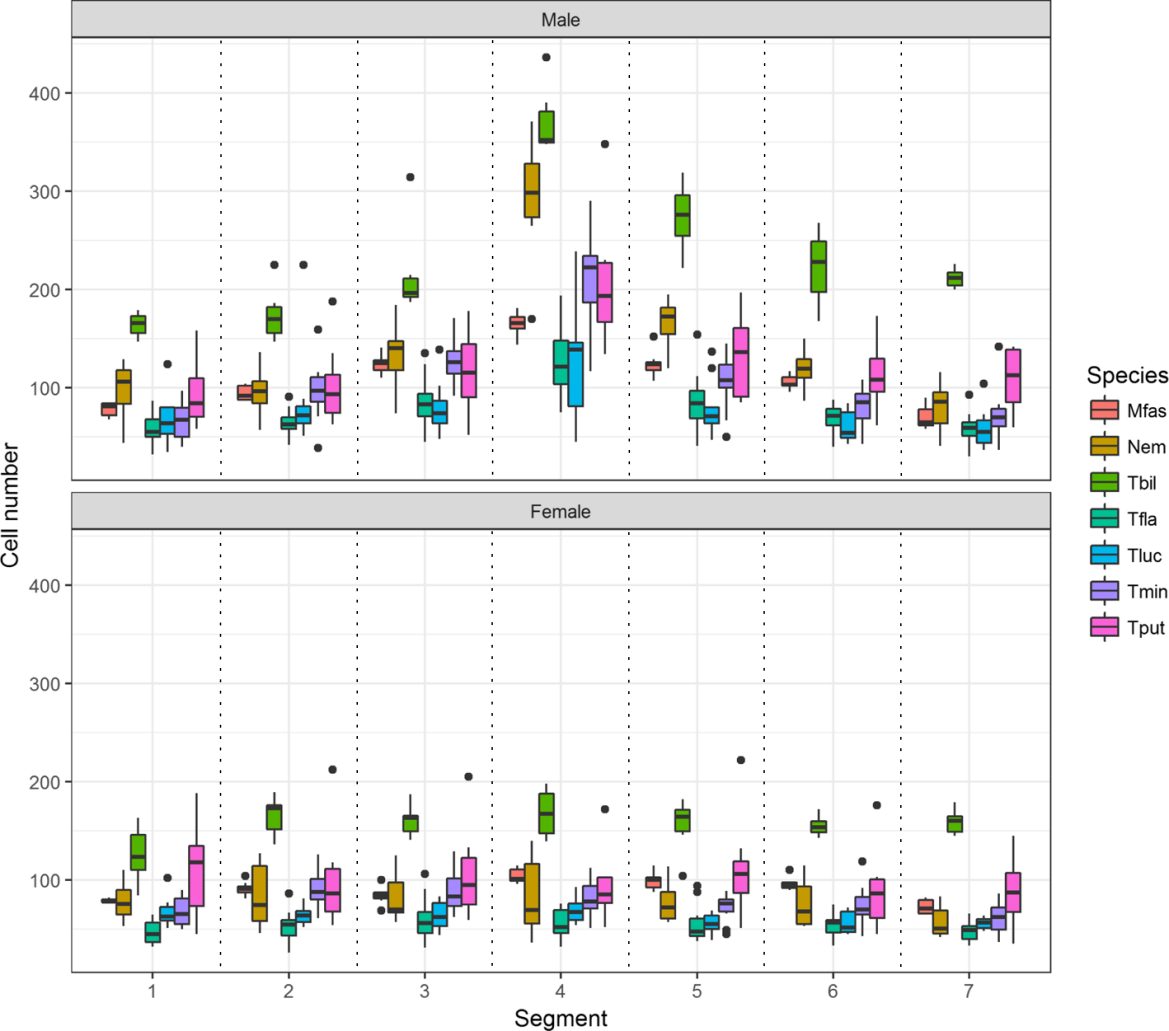
Primary gain of the appendage results in a strong sexual dimorphism and increase in cell number, nest size, and cell density within the nest in males peaking in the 4th abdominal segment which produces the abdominal appendage. Neighboring segments also show enlarged histoblast nests despite lacking the appendage. Females, which lack the appendage, show no significant difference in cell number or nest size between segments. Refer to Table 3 for the number of individuals sampled for each species

**Fig. 6:**
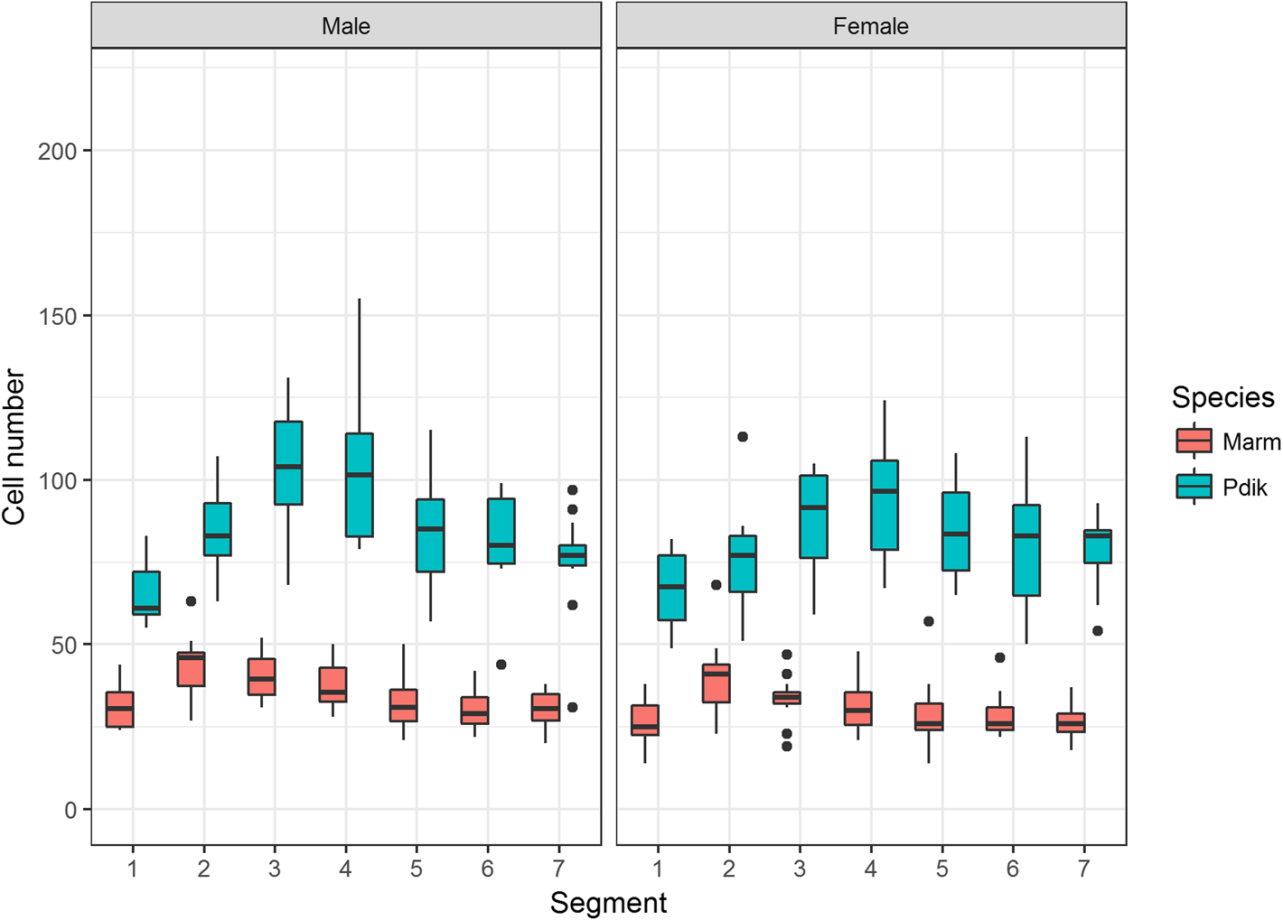
*M. armillata* and *P. dikowi* represent species with unusual histoblast nest patterns. Both possess the appendage, but nest morphology is not sexually dimorphic. *P. dikowi* females present the male phenotype having significantly enlarged nests in both sexes. *M. armillata* males present the female phenotype with no increase in nest size. *M. armillata* possesses diminished appendages and little sternite modification, indicating that small appendages do not require an increase in histoblast nest size. Refer to Table 3 for the number of individuals sampled for each species

**Fig. 7:**
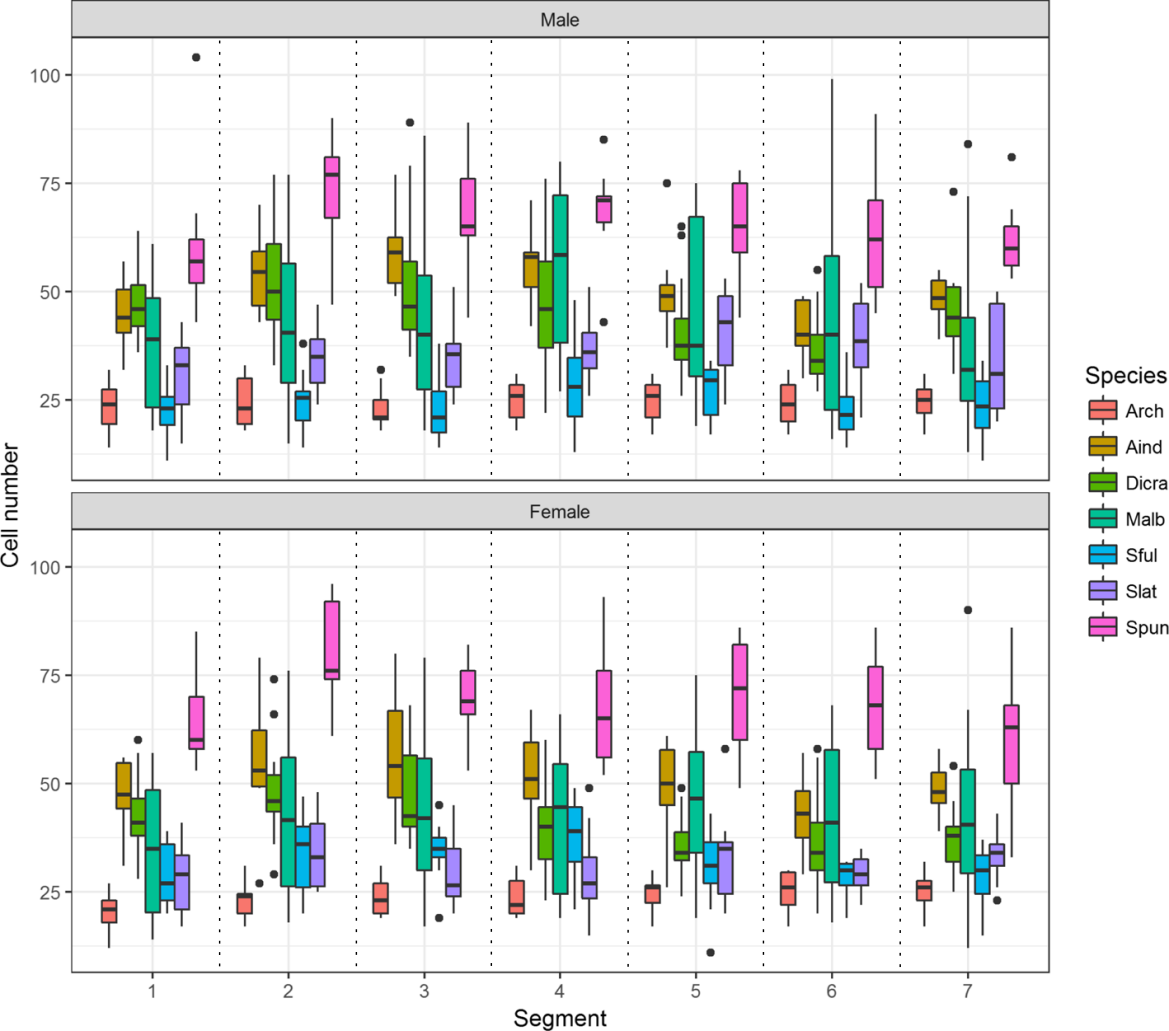
Secondary loss of the abdominal appendage results in a loss of sexual dimorphism. Nest size across segments remains significantly different but no consistent pattern exists between species. This group includes three independent losses with *A. indica, Dicranosepsis sp., S. latiforceps,* and *S. punctum* representing L1, *A. armata* representing L2, and *M. alberquerquei* representing L3. Refer to Table 3 for the number of individuals sampled for each species

Histoblast cell number was compared across sexes to determine if species were sexually dimorphic, using a likelihood ratio test (Table 1). The same test was used to determine whether the presence of appendages in the fourth abdominal segment correlated with an increase in histoblast cell number within each species. Three important patterns were revealed for species with appendages. Firstly, all species of *Themira, Nemopoda,* and *Meroplius* have a sexually dimorphic increase in histoblast nest size and cell count, which peak in the 4th abdominal segment (Fig. 5). Species such as in *T. biloba* and *N. nitidula* with both significant sex and segment effects indicate a pattern of histoblast nest morphology that is both sexually dimorphic and segment-specific. Secondly, *Perochaeta dikowi* has an increase in the 4th abdominal segment in both sexes (Fig. 6). This species does not have sexually dimorphic nest morphology because the female larva have enlarged nests in the 4th and neighboring segments which are not significantly different from males. Thirdly, *Microsepsis armillata* has no significant increase in histoblast cell number in the appendage-producing segment in either sex (Fig. 6). Species lacking the appendages due to secondary loss revert to sexually monomorphic histoblast nests without elevated cell number in the 4th abdominal segment. The only exception is *Meroplius alberquerquei* which lacks the appendage but has a sexually dimorphic enlarged nest in the appendage-producing segment (Fig. 7; Table 1).

**Fig. 8:**
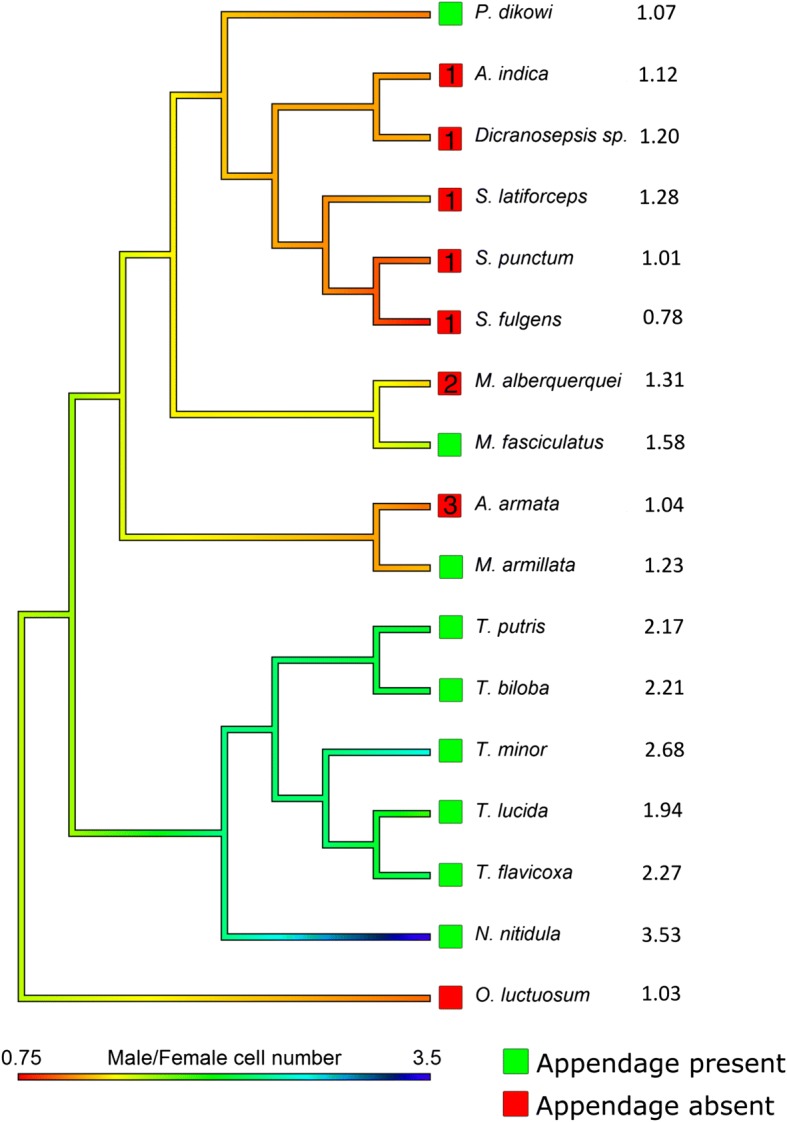
Phylogeny of sampled species indicating the degree of sexual dimorphism in histoblast cell number. Color of the terminal nodes indicates the ratio of male to female cell counts in the 4th segment histoblast nest with cooler colors indicating sexually dimorphic species. The value of the male:female ratio is reported. Colored boxes indicate whether the species has abdominal appendages (green) or does not (red). Independent losses are indicated as L1 – L3

In order to place the cell number in a phylogenetic context, we mapped cell number values onto the sepsid phylogeny. Using an existing sepsid phylogeny created using multiple genetic markers [18] we created a phylogenetic tree of the study species with colors representing the degree of sexual dimorphism in the mean 4th segment histoblast cell number with cooler colors representing increased dimorphism in the histoblast nests (Fig. 8). *Orygma luctuosum,* which lacks the appendage, has large nests and histoblast cell number but are sexually monomorphic in nest morphology. Species from the *Themira/Nemopoda* clade possess the appendage and have enlarged nests and elevated histoblast cell number that are both sexually dimorphic and vary significantly between segments (Tbil LRT, Sex = 73.69, Segment = 47.4, *p* < 0.001, Nnit LRT, Sex = 125.45, Segment = 67.86, *p* < 0.001) with *Nemopoda nitidula* and *T. biloba* possessing the greatest difference between the number of histoblast cells in the appendage producing segment of the species sampled (Fig. 5). *Perochaeta dikowi* possesses the appendage but lacks dimorphism due to enlarged female nests which are not significantly different from the male phenotype (Table 1). The same is observed for *Microsepsis armillata* which is the smallest species in overall size and has relatively small appendages with a sternite that is not as dramatically modified compared to the *Themira* and *Nemopoda* species. This may indicate that the degree of sternite modification is an important factor in histoblast nest size, but it also indicates that small sternite brushes do not require an enlarged histoblast nest. All species with putative secondary losses of appendages, also lose the sexual dimorphism in histoblast nest size.

### Cross species comparisons while controlling for phylogeny and body size

We compared histoblast cell counts across the entire data set to determine whether the presence of appendages was a predictor of histoblast cell counts in the fourth. Such comparison needs to account for differences in body size between species, and that closely related species are likely to have similar counts, independent of whether they have abdominal appendages. Sepsids show a large amount of variation in body size by species, and are frequently sexually dimorphic. Histoblast cell number is likely to be influenced by body size because histoblast cells form the abdominal epidermis in addition to the abdominal appendages [15]. Within our dataset, species with longer larval segments have an increased number of histoblast cells (Additional file 1: Figure S1). To test these relationships, we developed a linear model in which cell number was the dependent variable and sex (male vs female), length of each abdominal segment, and the number of that segment (1–7 treated as a categorical variable) were the independent variables. Sex (Z = 3.027, *p* = 0.0028) and segment length (Z = 8.766, *p* < 0.0001) both significantly contributed to cell number, indicating that males have more cells than females, and that larger individuals have more cells. Segment did not contribute significantly to the number of cells for any of the segments. However, Cook’s distance identified three cell counts that were significant outliers: the fourth and fifth segments of male *Themira biloba*, and the fourth segment of male *Nemopoda nitidula*. Interaction between sex and segment length was tested but was not significant, indicating that body size is not sexually dimorphic across the species sampled. This model fit the assumptions of normality, equal variance, and uncorrelated errors. The overall model explained 30% of the variation in histoblast cell number (F_(12,229)_ = 12.28, *p* < 0.0001, R^2^ = 0.3002). This model confirmed our hypothesis that body size, as approximated by segment length, is an important variable. Also, this model supported the conclusion that the specific segments are not contributing to the number of histoblast cells, with the possible exception of the fourth segment which contained two significant outliers.

Body size has a phylogenetic signal, with closely related species likely to have similar body sizes. Therefore, histoblast cell counts are likely to be influenced by phylogenetic relationships independent of the presence or absence of abdominal appendages. We used a generalized linear model to test whether the presence of abdominal appendages in males predicted an increase in histoblast cell number in the fourth segment while accounting for relatedness. A Markov chain Monte Carlo generalized linear model was developed in R using the packages ‘ape’ [19] and ‘MCMCglmm’ [20]. For the model, histoblast cell counts in the fourth male segment was the dependent variable and the independent variables were presence of abdominal appendages (N = no appendage, Y = appendage), segment length, and the interaction between the two variables. The phylogeny for the species (as determined by Su et al. 2016) was converted into a pedigree in which nodes of the tree were treated as parents to the species. The phylogeny was treated as a random effect. A Gaussian distribution was specified for the dependent variable. The priors for the random effect and the residuals were V = 1, nu = 0.02. The MCMC chain was run for 5 million iterations with the first 1000 iterations discarded as a burn-in and the remaining sampled every 500 iterations. Examining trace plots of the priors and posteriors indicated no autocorrelation between the variables.

The Markov chain Monte Carlo generalized linear model revealed a significant effect of the presence of abdominal appendages and histoblast cell count (pMCMC = 0.0024: Table 2). Having abdominal appendages meant more cells on the fourth abdominal segment even when accounting for phylogenetic relatedness and body size. The posterior for the random effect of phylogeny indicated significant phylogenetic inertia in the dataset (Table 2), confirming that it is important to control for relatedness in this analysis. In other words, histoblast cell counts are similar due to relatedness. Once phylogeny was controlled for, body size, as measured by segment length, was no longer a significant predictor of cell count (pMCMC = 0.2278), and the interaction between appendage presence and body size was not significant (pMCMC = 0.0626; Table 2). When the model was run without the interaction term, length remained not significant (pMCMC = 0.8692, DIC = 1623.268). Therefore, the presence of abdominal appendages predicts histoblast cell number across the phylogeny, independent of differences in body size.

**Table 2:** Effects on fourth segment histoblast cell count in male sepsids taking into account phylogenetic relationships between species conducted using MCMCglmm (DIC = 1620.037)

Effect	Posterior mean	Lower CI	Upper CI	pMCMC
Intercept	31.95	−61.8464	122.289	0.4853
Length	0.066	−0.0401	0.1739	0.2278
Appendage	197.8295	70.1738	325.2097	0.0024
Length: Appendage	−0.1692	−0.3548	0.003	0.0626
Phylogeny^a^	7528	2719	9998	

^a^Proportion of residual variance explained by phylogeny

## Discussion

The plasticity of the developmental processes that are involved in the formation of novel structures remain poorly understood. Here we provide new information on how developmental processes are related to morphological diversification of a novel trait, the abdominal appendages of some sepsid species. Addressing such questions requires comparing many closely related species that differ in the target phenotype [21]. The primary objective of this study was to identify patterns in histoblast nest morphology between segments, sexes, and species that are constrained by the evolutionary history of the appendage. The basic pattern of gains and losses had been previously described for *Themira biloba, Sepsis punctum,* and *Meroplius faciculatus* [14] and these species represented histoblast nest morphology in three clades that had distinct patterns in histoblast nest cell number. Here we intensively sample species closely related to those previously investigated to determine if these patterns represent distinct character states or if patterns of histoblast nest morphology are as variable across Sepsidae as abdominal appendage morphology. We also sampled species from three additional independent losses of the appendage and five additional genera to determine if species that lose the appendage return to the developmental mechanism of species that never had abdominal appendages. In addition, we studied larval segment length in order to control for the effect of organism size.

### The ancestral state

Based on previous data from multiple Diptera species and the information from the “basal” sepsid *O. luctuosum* presented here, we infer that the ancestral state for histoblast cell number is sexual monomorphism; i.e., a relatively consistent number of cells per segment across sexes. Histoblast cells have not been systematically sampled across Diptera, but the house fly, (*Musca domestica*) has approximately 200 cells per segment and *D. melanogaster* has smaller nests with approximately 15 cells per segment [16, 17]. In both species, cell number does not differ by segment or sex. In *O. luctuosum*, histoblast cell numbers are monomorphic across sexes, although the number of cells between segments is not consistent (Fig. 4). This is the likely ancestral condition for sepsid flies. There is a significant segment effect on cell number in *O. luctuosum* that was found in all other species with the exception of *A. armata* (Table 1) indicating that sepsids possess variation in histoblast nest morphology that is not found in *D. melanotaster* or *M. domestica*.

### Species with strict correlation between presence/absence of appendage and increase in histoblast nest size

The gain of abdominal appendages corresponds to an increase in the histoblast cell numbers in the male fourth segment (Fig. 5). This pattern of increase is consistent across eight of the nine species that have abdominal appendages. Larger species and species with large appendages and highly modified sternites have large nests. The *Themira/Nemopoda* clade contains species with the largest appendages and have approximately twice as many histoblast cells as *Meroplius fasciculatus*, a species similar in size but with much smaller appendages and sternites. These species exhibit a 3–4 fold increase in larval size over *Microsepsis* and approximately a 10-fold increase in histoblast cell number. Overall, the presence of abdominal appendages predicts an increase in nest size and cell number in the male fourth segment, even when body size and phylogeny are taken into consideration (Table 2).

Species without appendages have patterns similar to the ancestral state (Fig. 7). This suggests the loss of sexually dimorphic nest morphology and reduction in nest size and cell number accompanies a loss of the abdominal appendage trait. Depending on which character optimization is accepted, this pattern holds over two of the three independent instances of appendage loss. Five of the species without appendages show monomorphic histoblast cell counts, consistent with this pattern.

### Species with unusual patterns of histoblast nest size

Three species deviate from the overall patterns of a correlation between sexually dimorphic histoblast nest sizes and the presence of abdominal appendages. These species are particularly interesting because they allow for a better assessment to what degree histoblast nest evolution is required for the formation of abdominal appendages. The first exception is *Microsepsis armillata* which is the only species with abdominal appendages that lacks enlarged nests (Fig. 6). It has small appendages (Fig. 1i) and the histoblast nest size of the fourth segment is not enlarged. This indicates that the origin of appendages does not require an increase in histoblast nest size and that sexual dimorphism of sternites can entirely develop during pupation. Based on the position of this species in the sepsid phylogeny, the small histoblast nests in males are secondarily derived trait. This species illustrates both evolutionary plasticity and a developmental program that is flexible enough to produce novelty after histoblast nest specification.

The second exception is *Meroplius alberquerquei* which represents a loss of abdominal appendages in *Meroplius* (Fig. 3, L2). Yet, the histoblast nests remain sexually dimorphic. While *M. alberquerquei* is similar in body size to *M. fasciculatus* (Fig. 1f, g), it has much smaller nests. Some species that have lost the appendage have significant but non-sexually dimorphic differences between segments (Fig. 7). External morphology does not suggest any reasons for the non-sexually dimorphic differences in cell counts, however internal musculature also develops from histoblast cells and could be a possible source of the variation [12] given that there are distinct differences with regard to how extensively the abdomen of sepsids is using during mating behavior [10]. An alternative hypothesis is that early proliferation of histoblast cells is a trait retained from the common ancestor that did possess abdominal appendages. Although *M. alberquerquei* does not have abdominal appendages as an adult, we speculate that the loss has been so recent that early cell proliferation has been retained.

The third exception is monomorphic pattern of histoblast nest size in *Perochaeta dikowi*, a species that has abdominal appendages (Fig. 1a). Female *P. dikowi* have an enlarged 4th segment histoblast nest that is not significantly different from the male phenotype (Fig. 6). This is reminiscent of some female dung beetles that initiate growth of sexually dimorphic structures which are reabsorbed prior to becoming adults [22, 23]. Unfortunately, it is still not certain whether *Perochaeta dikowi* represents an independent origin of abdominal appendages in Sepsidae. Previous reconstructions of the sepsid evolution had favored this scenario [14], but under the most recent construction of the phylogeny, a parsimony reconstruction of appendage evolution implies that the appendage of *P. dikowi* is homologous with the one found in most genera (e.g., *Themira*).

An investigation of gene expression during embryogenesis and a comparison of gene expression between species in the developing appendage would provide insight in to how developmental mechanisms have diversified between species to produce these patterns of histoblast morphology and the diversity we see in the adult appendage. The genes *Notch*, *engrailed* and *extradentical* are expressed in the developing appendage in *Themira biloba* [24]. These genes have broad developmental functions, raising a potential for pleiotropic constraints. However, the current study suggests that histoblast proliferation, and by extension tissue remodeling, responds to selection with little evidence of constraint. Further investigations into gene expression in this tissue between species may reveal the source of this diversity.

## Conclusions

The evolution and diversification of novel traits in arthropods, especially in a family as diverse as Sepsidae, is not well understood. Using histology, we found that sepsid flies develop novel abdominal appendages from epidermal tissues that vary significantly between gains and losses of the appendage. This study supports the pattern that evolutionary gain of abdominal appendages usually corresponds to increases in cell number in the fourth segment in males, and loss of sexual dimorphism corresponds to a loss of this sexually dimorphic pattern in cell counts. This pattern persists across the Sepsidae family, even when body size and phylogeny are taken into account. However, some species show exceptions to this pattern, maintaining dimorphic cell number after the loss of appendages (*Meroplius alberquerquei*) and losing cell number dimorphism while still retaining abdominal appendages (*Microsepsis armillata*). The evolutionary changes in histoblast cells described here suggest a developmental system for mediating gain and loss that is labile and responsive to selection.

## Methods

### Species sampling

The most recent phylogeny of the Sepsidae supports a single gain of abdominal appendages and multiple independent losses [18]. Species were sampled across the Sepsidae to get multiple examples of species with and without abdominal appendages (Figs. 1, 3; Table 3). We sampled across the three putative losses of abdominal appendages, which we here designate as Loss 1, Loss 2, and Loss 3. Loss 1 is represented by Allosepsis indica, Dicranosepsis sp., Sepsis latiforceps, Sepsis punctum, and Sepsis fulgens (Fig. 1b-e). Loss 2 is represented by Meroplius alberquerquei (Fig. 1g) and Loss 3 is represented by Archisepsis armata (Fig. 1h). For each loss, we sampled an appendage-bearing species that was a close relative (Loss 1: Perochaeta dikowi; Loss 2: Microsepsis armillata; Loss 3: Meroplius fasciculatus). Species with abdominal appendages that were sampled included Nemopoda nitidula, Themira flavicoxa, Themira lucida, Themira minor, Themira biloba, Themira putris, Microsepsis armillata, Meroplius fasciculatus, and Perochaeta dikowi (Fig. 1a, f, i, j-n). Orygma luctuosum (Fig. 1o) was chosen because it lacks abdominal appendages and was expected to retain the ancestral developmental mechanism for forming sternites in males and females. Flies were obtained from live colonies at the National University of Singapore and North Dakota State University.

**Table 3:** Species sampled with number of individuals sampled and abdominal appendage status

Species	Males	Females	Appendage status
*Allosepsis indica*	14	13	Loss 1
*Archisespsis armata*	15	11	Loss 3
*Dicranosepsis* sp.	12	13	Loss 1
*Meroplius alberquerquei*	17	17	Loss 2
*Meroplius fasciculatus*	5	6	Gain
*Microsepsis armillata*	12	15	Gain
*Nemopoda nitidula*	8	6	Gain
*Orygma luctuosum*	8	7	Absent/Ancestral
*Perochaeta dikowi*	13	8	Gain
*Sepsis fulgens*	10	11	Loss 1
*Sepsis latiforceps*	17	18	Loss 1
*Sepsis punctum*	9	9	Loss 1
*Themira biloba*	6	6	Gain
*Themira flavicoxa*	14	11	Gain
*Themira lucida*	12	6	Gain
*Themira minor*	8	11	Gain
*Themira putris*	8	7	Gain

### Tissue collection

Larvae were fed ad lib on the preferred dung substrate of each species to ensure consistent age and nutrition at the time of collection. Flies raised at NDSU received a soy infant formula supplement solidified in agar under 1 cm of cow dung in a 15 cm petri dish. Dung was collected from a local organic cattle herd. Flies raised at NUS received the dung substrate preferred by that species. Duck dung was collected from the local poultry industry, and horse or cow dung was collected from the Singapore Zoo.

Third instar wandering-phase larvae were identified by their pale-yellow coloration, indicating gut purge and a commitment to pupariation. The wandering phase lasts for two to 4 hours across species. Sex of the larvae was determined by the presence or absence of testes, which appear as two large, clear ovoid masses between the 4th and 6th abdominal segments. Males and females were placed in separate collection dishes. Larvae were killed by immersion into water heated to 55 °C. This method causes muscle relaxation, which aids in dissection, and a consistent appearance of the larval epidermis. The larvae were dissected in an isotonic phosphate-buffered saline (PBS) 0.01 M solution. Lateral cuts at the location of the anterior and posterior tracheal cross-branches removed the head and open the larval posterior while leaving abdominal segments 1–8 intact. A longitudinal incision was then made along the dorsal line between the two main tracheal trunks. The tracheae, organ systems, and fat body were removed leaving a flat fillet of epidermal tissue.

### Tissue fixation and staining

Fixation and staining protocol was adapted from Madhaven and Madhaven. After dissection, tissues were rinsed in dissection buffer to remove remaining fat cells and transferred to a micro-centrifuge tube containing Kahle’s fixative (12% formalin, 32% absolute EtOH, 2% glacial acetic acid, 60% ddH_2_O) for 18–24 h at room temperature. Tissue was dehydrated in increasing concentrations of ethanol and stored in 100% ethanol at room temperature. Prior to staining tissue was rehydrated in decreasing concentrations of ethanol and finally in ddH_2_O.

Staining took place in a cell culture dish. First 2 mL of 6 N HCl was added for hydrolysis with 10-min incubation. HCl was removed and the tissue and well was rinsed several times to remove all traces of acid. 2 mL of Schiff’s reagent was added and the cell culture dish was placed in a light-proof box for 90 min at room temperature. After the 90-min incubation, the Schiff’s reagent was removed and replaced with distilled water and allowed to develop slowly under low light. After the desired level of stain was achieved the tissue was rinsed with distilled water several times to prevent further development. Every 30 min the distilled water was changed until it no longer colored pink. The tissue was stored in 100% ethanol in micro-centrifuge tubes prior to mounting.

Tissue was transferred to a glass dish containing 50% ethanol and Histoclear or HemoDe for 5 min after which it was replaced by 100% Histoclear or HemoDe. Each epidermis was immersed in mounting medium (PerMount) immediately prior to mounting. The flattened tissue was placed inside a drop of mounting medium and held in place by a weighted or clamped coverslip for 2–5 days while the mounting medium hardened.

### Histoblast imaging and characterization

Histoblast image acquisition was performed with an inverted Zeiss LSM microscope and Zeiss ZEN digital imaging software to generate z-stacked images of the histoblast nests (Fig. 2). The Schiff’s-stained histoblast nests fluoresce with little or no background fluorescence. Pixel dwell time was set to 1.11 s and image averaging was set to 2. These settings remained consistent across all samples, although the z-stack slice depth and window-size reflected the size of the nest between species. This information was preserved and accounted for during image data processing as part of the image file.

Histoblast nest nuclei were counted using the FIJI Image-J [25] package using a custom macro. The LSM z-stack images were flattened to produce a single TIFF image file that preserves scaling information. De-speckling, Gaussian, and median filters were applied to remove noise and the contrast against background was enhanced to resolve individual cell nuclei. An automatic local threshold was applied to convert the image to binary white nuclei on black background. A watershed tool was used to separate nuclei contacting other nuclei. The histoblast area of interest was then selected and an automated cell counter was used to collect the number of cell nuclei and the total, average, and individual area of the nuclei. FIJI was also used to measure total nest area by connecting the outermost nuclei at a point that includes all individual areas and measuring the internal space. Segment length was recorded using a calibrated measurement tool in AxioVision per segment per sample per species. Mean histoblast nest cell number per segment for males and females of sampled species are included as Additional file 2: Table S1. The complete dataset with measurements of histoblast cell number, histoblast nest area, and abdominal segment length both sexes and all sampled species are included as Additional file 3: Table S2.

### Statistical analysis

All statistics were conducted in R (version 3.3.3). A likelihood ratio test (LRT) was performed to determine significant differences between sexes and segments for each species using chi-squared values with the R package lmerTest. Linear models were made using the ‘lm’ function in R. The R package ‘phytools’ was used to generate and apply color values to a phylogeny based on trait values for all study species [26]. The Markov chain Monte Carlo generalized linear model was conducted using the packages ‘ape’ [19] and ‘MCMCglmm’ [20]. To control for phylogeny, relatedness was treated as a random effect in the model. The phylogeny was converted into a pedigree in which the “parents” of each species were the nodes of the phylogeny. The priors for the random effect and the residuals were V = 1, nu = 0.02. Histoblast cell counts in the fourth male segment was treated as continuous dependent variable, assuming a Gaussian distribution. The independent variables were presence of abdominal appendages (N = no appendage, Y = appendage) and the average length of the larval segments. Interaction terms were also tested. The MCMC chain was run for 5 million iterations with the first 1000 iterations discarded as a burn-in and the remaining sampled every 500 iterations. Autocorrelation was tested by a visual examination of trace plots of the priors and posteriors.

## Additional files

Additional file 1: **Figure S1.** The effect of mean segment length on the number of ventral histoblast cells per nest. Species with larger larval segments have more cells in the ventral histoblast nests. (PDF 27 kb)

Additional file 2: **Table S1.** Mean histoblast nest cell counts per segment for males and females of sampled species. (XLSX 12 kb)

Additional file 3: **Table S2.** The complete dataset used in this research containing measurements of histoblast cell number, histoblast nest area, and abdominal segment length for both sexes and all sampled species. (CSV 54 kb)

## Abbreviations

Aarm: *Archisespsis armata*
Aind: *Allosepsis indica*
Dicra: *Dicranosepsis sp.*
Malb: *Meroplius alberquerquei*
Marm: *Microsepsis armillata*
Mfas: *Meroplius fasciculatus*
Nnit: *Nemopoda nitidula*
Oluc: *Orygma luctuosum*
Pdik: *Perochaeta dikowi*
Sful: *Sepsis fulgens*
Slat: *Sepsis latiforceps*
Spun: *Sepsis punctum*
Tbil: *Themira biloba*
Tfla: *Themira flavicoxa*
Tluc: *Themira lucida*
Tmin: *Themira minor*
Tput: *Themira putris*
