# Supplementary figures and images for: Comparative analysis reveals the complex role of histoblast nest size in the evolution of novel insect abdominal appendages in Sepsidae (Diptera)
Source: BMC Evol Biol. 2018 Oct 10;18:151. doi: 10.1186/s12862-018-1265-3 (PMC6186081; doi:10.1186/s12862-018-1265-3)

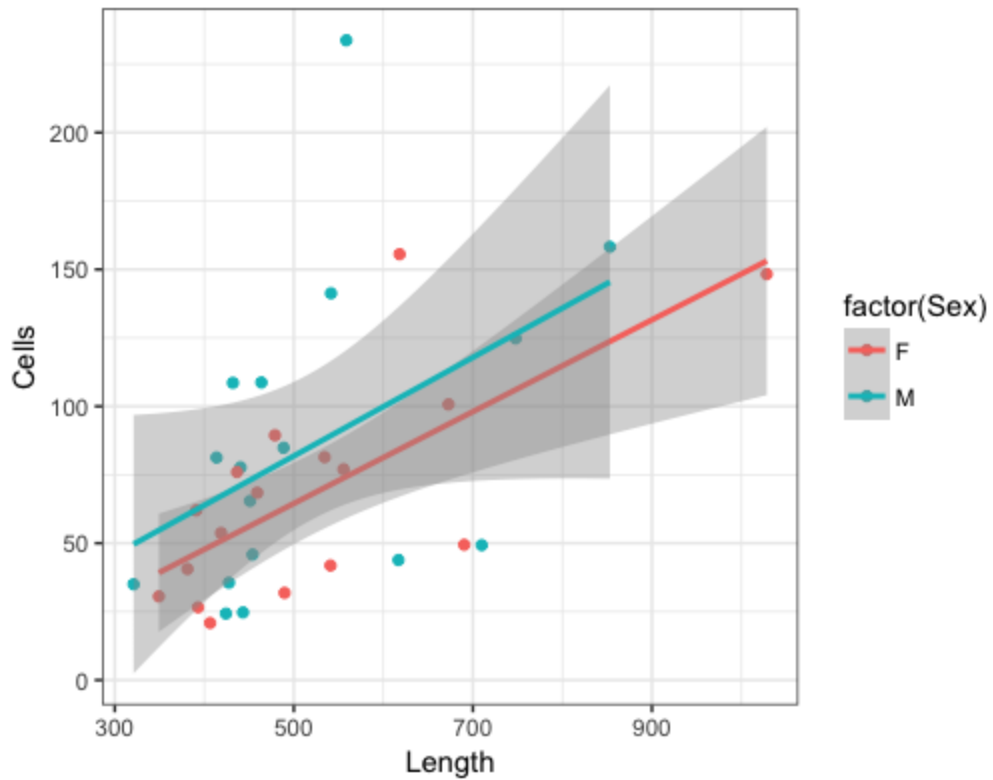

Supplement: Supplementary file 1 — Figure S1. The effect of mean segment length on the number of ventral histoblast cells per nest. Species with larger larval segments have more cells in the ventral histoblast nests. (PDF 27 kb) [file 12862_2018_1265_MOESM1_ESM.pdf]
